# Neonatal and carrier screening for rare diseases: how innovation challenges screening criteria worldwide

**DOI:** 10.1007/s12687-020-00488-y

**Published:** 2020-10-19

**Authors:** Martina C. Cornel, Tessel Rigter, Marleen E. Jansen, Lidewij Henneman

**Affiliations:** Clinical Genetics, Section Community Genetics, Amsterdam Public Health Research Institute, Amsterdam Reproduction and Development Research Institute, Vrije Universiteit Amsterdam, Amsterdam UMC, De Boelelaan 1117, Amsterdam, The Netherlands

**Keywords:** Neonatal screening, Genetic carrier screening, Rare diseases, Carrier state, Expanded screening, Treatment, Technology, Screening criteria, Reproductive autonomy

## Abstract

Screening for rare diseases first began more than 50 years ago with neonatal bloodspot screening (NBS) for phenylketonuria, and carrier screening for Tay-Sachs disease, sickle cell anaemia and β-thalassaemia. NBS’s primary aim is health gain for children, while carrier screening enables autonomous reproductive choice. While screening can be beneficial, it also has the potential to cause harm and thus decisions are needed on whether a specific screening is worthwhile. These decisions are usually based on screening principles and criteria. Technological developments, both treatment driven and test driven, have led to expansions in neonatal screening and carrier screening. This article demonstrates how the dynamics and expansions in NBS and carrier screening have challenged four well-known screening criteria (treatment, test, target population and programme evaluation), and the decision-making based on them. We show that shifting perspectives on screening criteria for NBS as well as carrier screening lead to converging debates in these separate fields. For example, the child is traditionally considered to be the beneficiary in NBS, but the family and society can also benefit. Vice versa, carrier screening may be driven by disease prevention, rather than reproductive autonomy, raising cross-disciplinary questions regarding potential beneficiaries and which diseases to include. In addition, the stakeholders from these separate fields vary: Globally NBS is often governed as a public health programme while carrier screening is usually available via medical professionals. The article concludes with a call for an exchange of vision and knowledge among all stakeholders of both fields to attune the dynamics of screening.

## Introduction

Technological developments have expanded the possibility to screen for rare diseases by increasing the availability of both treatments and tests in recent years. As a result, a larger number and broader range of diseases have been added to neonatal bloodspot screening (NBS) and carrier screening worldwide. While the expansion of screening could be beneficial, it also has the potential to cause harm. To balance screening benefits and potential pitfalls, policy makers traditionally developed screening programmes based on a set of defined principles and criteria. Rapid technological advancements, however, have shifted perspectives on screening for rare diseases and fuel debates on how screening criteria should be defined and used, both for NBS and carrier screening.

In this article, we argue that shifting perspectives on screening criteria for NBS as well as carrier screening lead to converging debates in these fields. To support our argument, we show how dynamics in rare disease screening have challenged four screening criteria: treatment, test, target population and programme evaluation. Screening criteria are usually based on screening principles, which we will introduce first. These criteria have evolved over time. Then, we summarize general aspects of NBS and carrier screening, followed by the dynamics in relation to the four screening criteria. We conclude by stressing the importance of an ongoing exchange of vision and knowledge between stakeholders involved in the governance of both NBS and carrier screening, as their expansion further converges their debates.

### Screening principles and criteria

Screening involves the medical examination of asymptomatic individuals for the early detection or exclusion of a disease. Screening is different from typical clinical care or diagnostics in that a test is offered unrequested. Early disease detection, before symptoms occur or are recognized, should do more good than harm (Raffle and Gray 2007). Therefore, in 1968, the World Health Organization (WHO) published “principles and practice of screening for disease” as a guideline to evaluate whether screening would be beneficial for a variety of conditions (Table 1) (Wilson and Jungner 1968). Screening *principles* clarify core concepts and values, while screening *criteria* define what it truly means to achieve and monitor the principle in practice. The Wilson and Jungner (W&J) principles for screening are still widely used, and are sometimes directly referred to as criteria. Many countries have operationalised criteria in different ways, tailored to their regional or national practice.

**Table 1:** Wilson and Jungner (1968) screening criteria and adaption of classical criteria by Andermann et al. (2008)

Wilson and Jungner (1968) screening criteria	
1. The condition sought should be an important health problem. 2. There should be an accepted treatment for patients with recognized disease. 3. Facilities for diagnosis and treatment should be available. 4. There should be a recognizable latent or early symptomatic stage. 5. There should be a suitable test or examination. 6. The test should be acceptable to the population. 7. The natural history of the condition, including development from latent to declared disease, should be adequately understood. 8. There should be an agreed policy on whom to treat as patients. 9. The cost of case finding (including diagnosis) should be economically balanced in relation to possible expenditure on medical care as a whole. 10. Case finding should be a continuing process and not a “once and for all” project.	
Adaption of classical criteria, by Andermann et al. (2008)	
1. The screening programme should respond to a recognized need. 2. The objectives of screening should be defined at the outset. 3. There should be a defined target population. 4. There should be scientific evidence of screening programme effectiveness. 5. The programme should integrate education, testing, clinical services and programme management. 6. There should be quality assurance, with mechanisms to minimize potential risks of screening. 7. The programme should ensure informed choice, confidentiality and respect for autonomy. 8. The programme should promote equity and access to screening for the entire target population. 9. Programme evaluation should be planned from the outset. 10. The overall benefits of screening should outweigh the harm.	

While the original W&J principles mainly discussed *whether* screening is worthwhile, revised versions of the W&J principles usually pay more attention to *how* screening programmes should be implemented. In recent screening frameworks, more attention has been placed on ensuring informed choice, equity and access, quality of care and cost-effectiveness (Andermann et al. 2008). Over the years, the W&J principles were not only translated into practical criteria, but policy advisory bodies have continued to adapt and expand these principles taking into account emerging technologies.

Forty years after publication, a revision of the W&J principles was published in light of advances in the field of genomics (Table 1) (Andermann et al. 2008). Moreover, screening criteria and ethical aspects specifically addressing genetic screening were published by various advisory bodies, for example the Nuffield Council on Bioethics (1993) and the Health Council of the Netherlands (1994). These publications highlighted the purpose of reproductive genetic screening in particular (Godard et al. 2003). In addition, while the original W&J principles were primarily aimed at finding disease at an early asymptomatic stage, screening criteria have expanded over time to include risk factors or susceptibility for a disease (Goel 2001), or to identify carriers who are at risk of having a child with a disease (Godard et al. 2003). The discussions in these publications illustrate how emerging technologies impact the dynamics of screening principles and criteria, which in turn impact the governance and operation of current screening programmes. In this paper, after introducing NBS and carrier screening, we focus on four of the criteria which have received attention in recent discussions (Grosse et al. 2006; Bombard et al. 2010; Wilson et al. 2019; Sinha et al. 2020; Modell 2020): treatment, test, target population and programme evaluation.

### Neonatal bloodspot screening

In NBS, diseases are identified early in life to offer timely intervention and improve health outcomes in children with rare, mostly autosomal recessive, disorders. In many countries, NBS began more than 50 years ago with screening for the genetic condition phenylketonuria (PKU) (Brosco and Paul 2013). Irreparable health damage can be avoided when infants with PKU are identified shortly after birth and prescribed a phenylalanine-restricted diet before the onset of symptoms. As a consequence of the clear health benefits, public health screening programmes were systematically organized in many nations worldwide to offer NBS to every infant (Therrell et al. 2015). Over the years, the programmes gradually grew and some now include more than fifty conditions (Therrell et al. 2015). Although PKU is a rare condition with 1–5 cases per 10,000 live births (orpha.net), PKU became a paradigmatic case for screening, effectively changing public health.

In their original publication, Wilson and Jungner (1968) explained why PKU is an important health problem: it is “extremely uncommon but warrants screening on account of the very serious consequences if not discovered and treated very early in life.” This is true for many of the diseases that are currently included in NBS programmes.

### Carrier screening

Genetic carrier screening identifies couples at high risk of having a child with a recessive disorder. It is estimated that there are more than 1800 recessively inherited rare diseases, with a range in symptoms from very mild to severe (Antonarakis 2019). When both partners are carriers of the same autosomal recessive disorder, couples have a 1-in-4 chance of having an affected child in each pregnancy. When the woman is a carrier of an X-linked disorder, there is a 1-in-2 chance for sons to be affected and a 1-in-2 chance for daughters to be carriers.

Approximately 1 in 100 couples faces such a risk globally (Ropers 2012; Haque et al. 2016). Some individuals in subpopulations have a higher than average risk based on geographic origin or (common) ancestry (Antonarakis 2019). Because carrier status generally does not affect one’s own health, most carriers are unaware of their status. As a result, the birth of an affected child typically occurs unexpectedly.

Some of the first carrier screening programmes involved Tay-Sachs disease in the Ashkenazi Jewish population in the USA, sickle cell disease in Greece and β-thalassaemia in Cyprus in the 1960–1970s (Kaback 2000; Antonarakis 2019; Delatycki et al. 2020). These programmes led to a marked reduction in the birth prevalence of these diseases. The often-cited primary aim was the prevention of rare, but severe, conditions in order to protect families and/or society from the burden of the physical, social and financial consequences (van der Hout et al. 2019). However, because of ethical tensions involving reproductive genetic screening, carrier screening was initiated in many countries with the aim to enhance reproductive autonomy and inform reproductive decision-making for (future) parents who have an increased risk of having an affected child (Cousens et al. 2010; Henneman et al. 2016; van der Hout et al. 2019; Antonarakis 2019). These options include not having (more) children, using a gamete (sperm- or oocyte-) donor, preimplantation genetic diagnosis (embryo selection), prenatal diagnosis with the option of termination if the foetus is affected by the condition and avoidance of marriage to another carrier (Godard et al. 2003). Availability and acceptance of these options vary due to cultural differences (Delatycki et al. 2020).

Unlike NBS, which is always offered shortly after birth, carrier screening is performed at different life stages. It can be offered to individuals or couples before pregnancy (preconception), before marriage (premarital or at high school) or prenatally. Preconception carrier screening may be preferable to prenatal carrier screening as it enables more reproductive options (Henneman et al. 2016). However, preconception carrier screening is not always feasible; for example, preconception care may not be systematically offered.

Carrier screening was not mentioned in the original W&J principles issued in 1968, which were primarily focused on detecting presymptomatic or early stages of a disease (Wilson and Jungner 1968). Carrier screening does not concern testing for an existing disease; rather, it aims to identify healthy heterozygous carriers who are at risk of having an affected child if the partner is also a carrier of the same disease. Furthermore, the reproductive choices at stake may be ethically sensitive, withholding jurisdictions to offer carrier screening as a public screening programme (van der Hout et al. 2019; Public Health England 2014). These two aspects make criteria other than the W&J criteria relevant in policy-making and implementation. Moreover, the ethical framework for reproductive genetic screening for rare diseases clearly differs from neonatal screening, as discussed below.

## Dynamics in screening criteria for rare diseases

In this section, we will discuss how dynamics in NBS and carrier screening have challenged four screening criteria (Table 1):

- There should be an accepted treatment for patients with recognized disease.
- There should be a suitable test or examination.
- There should be a defined target population.
- Programme evaluation should be planned from the outset.

In Table 2, we summarize the traditional interpretations and the relevant dynamics for these four criteria.

**Table 2:** Screening for rare diseases in neonatal screening and carrier screening—dynamics regarding four screening criteria. Dynamics in neonatal screening and carrier screening have challenged screening criteria. The traditional interpretations of the four criteria discussed in this paper and their dynamics are summarized in this table

Criteria	Dynamics	Neonatal	Carrier screening
There should be an accepted treatment for patients with recognized disease.	From treatment to actionability	Interventions (medication or diet) to avoid irreparable health damage innovate, i.e. “treatment-driven expansion”	Treatment in traditional sense not applicable, reproductive options available include preimplantation genetic diagnosis, prenatal diagnosis
There should be a suitable test or examination.	A suitable test transformed into a panel	From few to many conditions “test-driven expansion”	From few to many conditions “test-driven expansion”
There should be a defined target population.	The target population undergoes and/or benefits from screening.	Primary beneficiaries are newborns, but other beneficiaries are discussed, mostly families and sometimes society.	Individuals or couples considering a pregnancy or already pregnant; from screening “high-risk” populations (ancestry-based) to universal screening (pan-ethnic)
Programme evaluation should be planned from the outset.	Towards programme evaluation: matching the aims	Evaluations should include follow-up on health outcomes of infants.	Evaluation should include measure of informed choice.

### From treatment to actionability

For NBS, “an accepted treatment for patients” is a criterion for disorders to be included in the screening programme. In the W&J principles, it refers to an intervention that ensures health gain and reduction of mortality and morbidity (Wilson and Jungner 1968). As a consequence, when a treatment becomes available and/or acceptable for a disorder that causes serious health problems, it becomes eligible for inclusion in the NBS panel. The number of treatable hereditary disorders is increasing rapidly, especially for rare diseases. Recent years have seen a strong technology push, both in treatments and tests. For example, a significant technology push is coming from stem cell transplantation and enzyme replacement therapies. Moreover, gene therapies are on the horizon and may significantly increase the number of treatable rare disorders. Treatment innovations are illustrated by the recent inclusion of severe combined immunodeficiency (SCID) in a number of NBS programmes. SCID is the first NBS disorder that does not include a lifelong intervention regimen to prevent irreversible health damage; instead, it is cured by an available treatment (Fischer et al. 2015). Also, for spinal muscular atrophy (SMA), a disorder considered for inclusion in NBS panels, a cure might be available in the foreseeable future with gene therapy (Mendell et al. 2017), albeit at a very high financial cost.

The treatment-driven expansion fuelled by treatment innovations requires careful decisions from policy makers about the inclusion of more disorders in NBS panels. The “treatability” screening criterion has received much attention in these debates (Bombard et al. 2010): What reduction of mortality and morbidity is required to be marked as an acceptable treatment for patients with recognized disease? In NBS, this discussion is complicated by the rarity of included disorders. Scientific evidence regarding the effectiveness of treatment for these small groups of patients is often limited. As a consequence, the level of evidence that countries use to assess an acceptable treatment often influences the decisions regarding the NBS panel composition (Jansen et al. 2017; Mackie 2017).

Not only does the available scientific evidence of a treatment influence the disorders included, but also the applied definition of health gain. Benefits of screening other than health gain might be relevant when debating disorders that are currently marked as lacking effective interventions. For example, early diagnosis of Duchenne muscular dystrophy (Ross and Clarke 2017), or ataxia telangiectasia, an untreatable incidental finding in SCID screening (Blom et al. 2019), might prevent a diagnostic odyssey involving many hospital visits and unnecessary, sometimes harmful, tests. The ability to identify carrier status in neonates after NBS is also sometimes suggested as a benefit, because it can inform subsequent reproductive decisions for the child in adulthood. In addition, if carrier status is detected in the newborn, at least one parent is most likely a carrier as well and this knowledge will inform future reproductive choices for the parents (Bombard et al. 2010). For founder mutations, esp. haemoglobin disorders in populations of African or Mediterranean descent, there is an increased likelihood that the other parent is a carrier as well, and a subsequent child may have a severe haemoglobinopathy. Therefore, countries need to consider carefully if and how haemoglobinopathy carrier status results from NBS are communicated (Burgard et al. 2012).

Whether these additional screening benefits (avoiding a diagnostic odyssey, informing couples about risks for future pregnancies) meet accepted screening criteria remains debatable, as they directly influence the aim of NBS, and other options such as carrier screening could be a suitable alternative.

The criterion of “acceptable treatment” in carrier screening deserves special attention, as the goal is not treatment in its traditional sense, but involves reproductive autonomy. It might be more appropriate to speak about “acceptable intervention” or “acceptable care”. As outlined above, one of the options for carrier couples is prenatal diagnosis. Pregnancy termination of an affected foetus is sometimes described as “therapeutic abortion”, but others argue that abortion can never be considered a treatment. The criterion needs to be broadened to: “there must be ‘practical courses of action for participants’ … because this decision can only be justified as the highly personal choice of the pregnant woman (and her partner), the term ‘prevention’ must be used very cautiously in this context. Selective abortion is not a normal preventive measure, and must not be presented as such.” (Health Council of the Netherlands 2008; Van El et al. 2012).

The ethical framework used in genetic counselling is therefore considered more suitable in evaluating the aspect of “acceptability of intervention” in reproductive screening. Helping and empowering couples to make an autonomous informed choice are important ethical principles in this context (American Society of Human Genetics 1975). The concept of non-directiveness provides an appropriate framework for genetic counselling, especially in relation to reproductive decisions (Elwyn et al. 2000): The main question is whether the intervention is acceptable for the couple involved.

### A suitable test transformed into a panel

The W&J criterion states that there should be “a suitable test” available. The first NBS test developed for PKU by Dr Robert Guthrie was a bacterial inhibition assay (Blumenfeld et al. 1966). Several NBS tests used in the following decades assayed specific metabolites and enzyme activity for disease detection. A technology push was already seen in the 1980s with the development and use of tandem mass spectrometry (MS/MS) (“test-driven expansion”). Unlike blood analyses that could be used for one condition, MS/MS enabled the inclusion of many metabolites in one test, supporting the simultaneous testing of many metabolic diseases in NBS (Therrell et al. 2015).

Another innovation that could significantly push test-driven expansion is the use of DNA-based testing, for example, through sequencing technologies. DNA-based testing in NBS developed later than biochemical testing. It is commonly used as a follow-up test after biochemical testing in NBS for cystic fibrosis (CF) or to quantify T cell receptor excision circles in SCID. Now that less expensive high-throughput sequencing is becoming widely available, the possibilities of DNA-based NBS for an increasing number of conditions are being actively discussed (Howard et al. 2015; Johnston et al. 2018). Not only can DNA technologies enable testing for new NBS conditions such as SCID and SMA, it can also be used to minimize false-positive screening results as is the aim for CF. However, DNA testing can also lead to the identification of DNA variants that are less pathogenic (e.g. CFTR p.Arg117His) or variants of unknown significance, a potential drawback of NGS applications in screening. Application of a mixture of biochemical and DNA-based methods may result in the best trade-off of sensitivity vs. specificity.

Test-driven expansion for carrier screening has been very similar to the progression seen in NBS testing. While in the previous century, high-performance liquid chromatography (HPLC) and electrophoresis were available for haemoglobinopathy screening, and carrier status of Tay-Sachs disease was screened based on hexosaminidase A enzyme activity, currently next-generation sequencing enables carrier screening for many conditions simultaneously (Edwards et al. 2015; Henneman et al. 2016). Although professional guidelines dictate that disease severity is a key criterion for offering carrier screening (Edwards et al. 2015; Henneman et al. 2016), it is a matter of debate which disorders should be included and how to define the severity of disease (Lazarin et al. 2014). In contrast to NBS public health programmes, the expansion of panels in carrier screening is currently mainly driven by commercial interests and not based on professional guidelines or defined criteria, resulting in a wide variety of tests covering hundreds of conditions (Chokoshvili et al. 2018).

Besides the innovation in testing methods such as MS/MS and DNA technologies, bioinformatics can also stimulate expanded screening for rare diseases or improve current panels by minimizing false positives. Both MS/MS-data and DNA data potentially include more information than what is currently analysed. For instance, bioinformatics may enable reporting of (likely) pathogenic mutations for recessive diseases by analysing a multitude of gene variants using large reference databases. Also, the many informative markers produced by MS/MS can be integrated into new algorithms that calculate the posterior probability of certain rare diseases, thus improving the predictive value of the analysis. Post-analytical interpretive tools can integrate all relevant results into a single score (Hall et al. 2020). These developments in bioinformatics could greatly impact both NBS and carrier screening.

### The target population undergoes and/or benefits from screening

Concerning the target population of screening, Wilson and Jungner (1968) mentioned as a principle that it should be clear who to treat as a patient, while Andermann et al. (2008) broaden this to include the target population as a whole. The target population in this regard refers not only to the population undergoing the test, but also to the population that is most likely to benefit from screening. This can be the individual, the family and/or society. To justify NBS, interpretation of screening criteria traditionally focuses on the health gain for the newborn population. In NBS, different perspectives are seen in policies regarding the beneficiaries of screening (Jansen et al. 2017). As illustrated before, other benefits are sometimes used in arguments to include certain diseases in screening panels. Often these arguments focus not only on *what* the benefit is—and if it can be considered acceptable as a health gain—but also on *who* receives this benefit. The family can be considered the beneficiary when, for example, a diagnostic odyssey is reduced or prevented, limiting psychosocial consequences for both the child and the family. Moreover, due to the time span of a diagnostic odyssey, another child with the same disorder may be conceived, which would result in two children with the same condition, heavily burdening the family.

As discussed previously, the main aim of carrier screening is to offer informed reproductive decision-making. Consequently, couples considering a pregnancy or those already pregnant are the key beneficiaries of carrier screening. However, in the light of increasing costs for care and treatment of patients with rare diseases, which can strain (health care) budgets within certain communities that have a relative high frequency of specific recessive diseases, some argue that societal impact may be seen as a justified argument to implement carrier screening (de Wert et al. 2012; Sinha et al. 2020). In that case, society becomes a beneficiary through reduced disease burden and increased knowledge about rare diseases. The *Journal of Community Genetics* recently outlined a situation in India where the increased life expectancy for patients with sickle cell anaemia and β-thalassaemia led to an “imperative for the Government of India to devise cost-effective preventive strategies to reduce the burden of disease”, especially by carrier screening (Sinha et al. 2020). In an accompanying editorial, Modell (2020) described the dilemma as “how to commit to providing optimal care when it may not be affordable in the long term”, and also referred to the UK, where lifelong treatment for CF patients was not approved on the grounds of unaffordable cost. In Mediterranean countries where prospective β-thalassaemia carrier screening was introduced decades ago, the birth prevalence of haemoglobin disorders fell substantially making optimal care for patients affordable.

Historically, carrier screening programmes targeted specific diseases in well-defined, high-risk populations. Expanded carrier screening is expected to increase access to screening opportunities for people who do not belong to a high-risk population (Edwards et al. 2015), thus expanding the target population. A pan-ethnic or universal offer that allows testing of all individuals regardless of ancestry has the potential advantage of reducing stigmatization of ethnic groups (Henneman et al. 2016). Notwithstanding these advantages, ethical concerns remain an issue (van der Hout et al. 2017). One concern is that expanded carrier screening may confront prospective parents with complex decisions and dilemmas (van der Hout et al. 2017). On the societal level, the proportionality of large-scale screening implementation needs to be assessed: Does the value of expanded carrier screening justify the use of limited health system resources (Wilfond et al. 2018)? Concerns also exist regarding the possible impact of expanded carrier screening on people affected by genetic conditions, including a reduction of societal support, medical expertise and research funding associated with the declining numbers of people born with specified genetic conditions (Boardman and Hale 2018).

### Towards programme evaluation: matching the aims

Another new criterion that emerged with the revision of screening criteria by Andermann et al. (2008) is that screening programme evaluation needs to be planned from the outset (Table 2). As a consequence, a programme may be modified or even discontinued based on ongoing assessment (Andermann et al. 2010; Wilson et al. 2019). Guidelines for evaluation include feedback from a diagnostic unit to a screening laboratory, which enables evaluation of basic aspects like prevalence, sensitivity and specificity. A critical, but more complicated aspect of evaluation is appraising the outcome of a programme. For example, the primary objective of neonatal screening is health gain for the newborn; therefore, one would expect that this is a main evaluation outcome. However, determining health gain requires evaluating the quality of life over several years. Although many programmes have increased their efforts to gain insight into the long-term results of screening, this has not proven easy. For instance, registry-based evaluation of long-term outcomes is almost non-existent (Burgard et al. 2012). Moreover, if the (secondary) beneficiary of NBS is the family or even society, they should be included in the evaluation, which is not usually the case.

Similarly, the evaluation of reproductive carrier screening efforts should be tailored in order to measure whether the initial goals are being met. As discussed above, in some cases, prevention is considered a justified goal of reproductive carrier screening. Programmes aimed at prevention have been explicitly evaluated in terms of the decline in the number of children born with the disease (Laberge et al. 2010). However, if the aim of reproductive carrier screening is to allow for autonomous reproductive choice, then the evaluation of the screening should assess to what extent informed decision-making takes place. This has also been argued for prenatal screening for foetal abnormalities (Dondorp et al. 2015). In this case, the goal (to facilitate informed choice) should thus be distinguished from the possible consequence (a reduction in live birth prevalence) (Ten Kate et al. 2010). Measuring informed choice has, however, proven difficult, especially since there seems to be no consensus on the best methodology for the evaluation of informed decision-making. To shed more light on how screening results impact the reproductive choices of carrier couples, studying longer-term outcomes can provide helpful insights (Cannon et al. 2019), but similar to neonatal screening, it is not easy to follow these couples over time.

## How innovations make debates converge

We have discussed how innovations in tests and treatments drive changes in NBS and carrier screening, leading to debates about the target population and beneficiaries. From these discussions, it is apparent that when the (goal of) screening changes, so should its evaluation.

If innovations lead to an effective intervention for a disease, it could become suitable to include it in NBS programmes. Subsequently, treatment success should be evaluated and it may be assessed whether in the long-term preconception testing has become obsolete. For example, if a treatment for SMA could treat or even cure infants at an affordable price, it should be included in NBS (Health Council of the Netherlands 2019; Mendell et al. 2017), and preconception carrier screening for SMA would perhaps no longer be needed. However, the reverse argument was made recently: If a treatment becomes available that increases the societal and especially economic impact of a disorder, this may become a burden for society, thus making preconception carrier screening even more urgent (Sinha et al. 2020). This resembles the arguments on carrier screening for ß-thalassemia in Cyprus in the early1970s (Delatycki et al. 2020).

Because of technological developments in testing, more disorders are included in screening programmes. As panels are expanding, so are the aims of screening. In carrier screening offers, treatable disorders are now included for which earlier recognition (i.e. before NBS) could improve health outcomes, for example medium-chain acyl-CoA dehydrogenase deficiency (MCADD). Here, the aim of carrier screening is not to inform reproductive decision-making, but to achieve an earlier diagnosis and provide early therapeutic options, i.e. before the result of NBS becomes available. In neonatal screening, conditions are included for which insufficient data are available for a strict evaluation according to current principles, but allow an early diagnosis, which in turn can benefit future family planning and research. Testing per condition becomes cheaper when the same technology is used for many conditions, but the evaluation of clinical validity, clinical utility and ethical, legal and societal issues (ELSI) may differ between conditions. Using one test for many conditions (almost), for the same price, may look attractive, but without the careful weighing of risks and benefits, the programme may inadvertently start to include conditions which mainly are symptomatic in adults, or variants for which the penetrance is not completely understood and for which the advantages of early treatment are unclear. Thus, the technology push can also adversely alter the balance of advantages and disadvantages of NBS.

Screening in one phase of life often impacts other phases of life. If NBS identifies a newborn with CF, preconception carrier screening of *those* parents is no longer needed as they are identified as obligate carriers. In Israel, because of a nationwide CF carrier screening programme, fewer children—and with relatively milder phenotypes—are born with CF and policy makers therefore decided not to include CF in the NBS panel (Stafler et al. 2016). However, carrier screening does not replace NBS, nor does NBS diminish the potential benefit of carrier screening (ACOG 2017). NBS is still important, as often a screening test will not detect all carriers, and not all prospective parents will decide to participate (Stafler et al. 2016) or act on their screening results (Cannon et al. 2019).

The stakeholders who organize, coordinate, offer and evaluate screening differ between NBS and carrier screening. While NBS is often created and governed as a national public health programme, initiators of carrier screening vary globally, from governments and communities to commercial providers, and are increasingly offered in an ad hoc manner by (individual) physicians. However, while the same expertise is usually needed in decision-making and implementation of both NBS and carrier screening, the stakeholders involved are often quite different: public health experts vs. medical specialists, laboratory experts vs. private companies. Especially with the convergence of the debates, it would be appropriate to exchange views between stakeholders involved in both, or even aim to have the same experts at the table.

## Conclusion

The fields of neonatal screening and carrier screening are dynamic, and the interpretation and application of screening principles and criteria evolve over time. This requires constant evaluation and ongoing interdisciplinary debates on the interpretation and application of screening principles. Well-defined screening aims should be established and maintained to ensure adequate programme evaluation on these aims is in place. Technological innovations enable rapid treatment—and test-driven expansion for rare diseases, which could make a disease simultaneously considered for neonatal as well as carrier screening. Both the treatment-driven and test-driven technology push lead to a need for an alignment of aims and evaluation, involving all relevant stakeholders. To facilitate attuning dynamics within both neonatal and carrier screening, not only is transparency about decision-making required, but an exchange of vision and knowledge between all stakeholders from both fields is imperative.
